# Remarks on Schools of Instruction for Military and Naval Surgeons, in a Letter to the Right Hon. Sir Robert Peel, Bart

**Published:** 1844-04-01

**Authors:** 


### Remarks on Schools of Instruction for Military and Naval Surgeons, in a Letter to the Right Hon. Sir Robert Peel, Bart. By Sir *George Ballingall,* M.D., Regius Professor of Military Surgery in the University of Edinburgh.

The subject of medical education has of late deservedly received much consideration, and various modifications have been introduced by the different colleges into the course of study required of candidates for their diplomas. Our attention has been directed, by the pamphlet now before us, to the qualifications demanded of those who are ambitious of serving in the medical departments of the Army and Navy, and to the means provided for their special instruction. It is undoubtedly the imperative duty of Government to supply efficient medical assistance, by means of a properly qualified body of officers, to those engaged in the protection of British interests in our widely extended possessions, where they are frequently exposed to those causes of sickness and mortality which decimate the ranks of an army far more surely than an enemy's bullets, and may overturn the best concerted plans of military operations. To fulfil this obligation, it would appear necessary to secure the services of individuals who have devoted themselves to the study of the various branches of medical science, and are well versed in its general principles. But while we assume that a knowledge of these principles and of their practical application, is sufficient for the successful treatment of disease in any quarter of the globe, the question may still be asked, is there any peculiarity in the condition of the soldier or sailor which requires a different course of study from that necessary for the surgeon destined for practice in civil life? This opinion is held by our author, the object of whose pamphlet seems to be, to show "the liberal provision made by some foreign states, particularly Prussia and Austria, for the education of their Army Surgeons, compared with the very scanty provision made for the same purpose by our Government," and to recommend "the endowment of a professorship of military medicine and surgery in London, and another in Dublin."

We may remark that the liberality on the part of these Governments (if it deserves that name), is in some measure rendered necessary by the niggardly hand with which they dole out pay and retiring allowances to the medical officers. These are framed on so economical a scale, that few persons in a position to educate their sons at their own expense consider the army to hold out sufficient inducements to place them in it, and the Government is consequently obliged to obviate the want of candidates resulting therefrom, by contributing liberally towards the training of individuals for this department. In the British service however the remuneration, present and prospective, is calculated more equitably, and no difficulty is experienced in procuring a sufficient number of well-qualified officers without any such assistance.

Our author considers a course of lectures on military surgery to comprehend "all that in either branch of the service (Army or Navy) contributes to the prevention as well as the cure of disease," justly observing, "it cannot be too often repeated, that it is by prevention rather than by cure, that the efficiency of our fleets and armies is to be maintained." He states it as his opinion, that the only men qualified to fill the proposed professorships with advantage, are "those who have gained experience abroad in the service of their country, and who consequently have established a claim on the public to half-pay." By this it is doubtless intended that medical officers of either branch of the public service should be deemed eligible, although nothing is expressly stated in the letter on this head; but it need scarcely be observed, that an Army-Surgeon may be very imperfectly acquainted with the economy of a man-of-war, and therefore by no means qualified to deliver a course of lectures on naval hygiène; while on the other hand, a naval surgeon, however well-informed in regard to his own branch of the service, may not be very competent to instruct medical officers of the Army respecting the best means of preserving the health of soldiers.

In recommending the establishment of two additional professorships of military surgery, Sir George Ballingall intends, we presume, that this should become a compulsory class, and that every candidate for admission into the medical department of the army, should be obliged to fee one or other of these professors. Has he assigned adequate reasons for this important measure? We think not. Such a regulation might justly be considered a hardship, as a very small proportion of the candidates ultimately obtain the object of their wishes. An endowment is no doubt an excellent thing for the endowed; but has he shown that the curriculum of study for the Army or Navy is inadequate? Are the medical officers of either service not sufficiently conversant with their duties? Is the discipline young officers undergo at Fort Pitt or Haslar, and in a regiment or on ship-board, not adapted to initiate them into the mysteries of their respective appointments? What practical advantages have resulted, during the last 30 years, from the establishment of a chair of Military Surgery in Edinburgh? Are the few who have attended this class more efficient, more studious, better informed, more accurate observers, or more zealous and industrious in collecting and diffusing the results of their experience and observations than their brother officers?

With respect to the elementary and professional education which a candidate for admission into the Army must possess, it may be briefly stated, that a competent knowledge of the Greek and Latin languages is indispensably requisite, and no one can be appointed who does not possess the diploma of the Colleges of Surgeons of London, Edinburgh, or Dublin, and who cannot produce testimonials of—

| | | |
|---|---|---|
| 18 | months, | Hospital Attendance. |
| 24 | ,, | Anatomy. |
| 12 | ,, | Practical Anatomy. |
| 12 | ,, | Surgery. |
| 8 | ,, | Clinical Surgery. |
| 12 | ,, | Practice of Physic. |
| 8 | ,, | Clinical Lectures on ditto. |
| 12 | ,, | Chemistry. |
| 6 | ,, | Practical Chemistry. |
| 3 | ,, | Botany. |
| 4 | ,, | Materia Medica. |
| 3 | ,, | Practical Pharmacy. |
| 5 | ,, | Natural History. |
| 5 | ,, | Natural Philosophy. |
| 3 | ,, | Midwifery. |

A similar course of study is required of candidates for the Navy, and will be generally allowed, we believe, to be sufficiently extensive; indeed it is more so, both in regard to the number of classes and the period of attendance enforced on several of them, than that of any of the licensing bodies in the United Kingdom.

Military Surgery, as has been already stated, comprehends the study of the prevention as well as the cure of diseases, consequently, in a course of lectures on this subject, hygiène may be expected to occupy an important place. The various topics to be discussed under this head, are given at great length by Vaidy in his article "Hygiène Militaire," in the Dictionnaire des Sciences Medicales. The more important of these are thus briefly enumerated by Mr. Marshall.*

"The recruiting of the Army, bounty, pay, pensions, rewards; provisions, messing, &c.; barracks, transports, and clothing; personal cleanliness; duties and exercise of soldiers; schools and regimental libraries; military discipline, punishments, coercive and corporal; habits of soldiers, comprehending virtues and vices;—the constitution of the medical department, and the duties of the medical staff, both general and regimental; military hospitals, moral treatment of the sick, the compilation of numerical returns of the sick, and the plan of drawing up reports of diseases, both general and special;—proceedings of boards, sick certificates, &c., together with the general principles of military statistics and medical topography; and lastly, instructions to young medical officers, respecting their general conduct, especially in regard to their superiors, their equals, inferiors, and patients."

We beg our readers not to conclude, that because, in our opinion, Sir George Ballingall has failed to make out a case for the endowment of two new professorships, we deem the careful study of the means of preserving the health of soldiers—military hygiène—unimportant. We have quoted above, the leading subjects embraced by it, in order to shew to those unacquainted with the subject that it is of the first consequence; but it is

* Edinburgh Medical and Surgical Journal, No. 124.

one of those branches of knowledge which may be acquired as well in the closet as the class-room. It is true there is no comprehensive work on the hygiène of the British Army and Navy,—one which treats in detail of the various duties of medical officers in these branches of the public service; but the more important facts may be acquired from separate treatises. We may particularly mention Millingen's Army Medical Officers' Manual—Hennen's Military Surgery and Medical Topography of the Mediterranean—Dr. Jackson on the Formation, Discipline, and Economy of Armies—Ballingall's Military Surgery—Marshall on the Enlisting and Discharging of Soldiers—and the Statistical Reports on the Health of the Army and Navy. If we are rightly informed, the Director-General of the Army Medical Department at one time contemplated a treatise on military hygiène, and had made some progress in its execution, but whether it be near its completion or likely soon to be published, we are ignorant. A work of this kind from authority, would, we think, be of much advantage to the service; indeed, a comprehensive treatise on the best means of preserving the health of soldiers, by a talented, industrious, experienced medical officer, although of subordinate rank, might convey much useful information to the junior members of the department.

Among the reasons advanced in favour of this scheme of endowment, is the necessity of having some one to explain and comment on the "Statistical Reports on the Sickness, Mortality, and Invaliding among the Troops," and the "Statistical Reports on the Health of the Navy." "These have been prepared," says Sir George, "at an enormous amount of labour to the authorities at the Horse Guards, the Medical Board and the Admiralty, and at a very considerable pecuniary expense to the public. All this labour, all this expense will, I confidently assert, be in a great measure lost to the service, unless means are taken to concentrate the valuable information embodied in these reports, to put it in an attractive form, and to keep it constantly before the present and rising generation of army and navy surgeons." Now, in adducing this as an argument, we think our author has been rather unfortunate; for we have always considered these Reports to be so remarkably plain and unambiguous, and the conclusions deducible so clear, as to be perfectly intelligible to the meanest understanding. They are, moreover, works which ought not to be taken up merely as the groundwork of a few passing observations in a lecture-room, and then be thrown aside, but are worthy of repeated careful perusal, and on foreign stations ought to be books of constant reference. This view appears to be taken by Government, as copies have been transmitted to every regiment, to be retained as official documents, and we have reason to believe they have been more justly appreciated by officers on foreign service, who could not have availed themselves of the advantage of having them expounded *ex cathedra*, than they have been in this country.

Our author has long been distinguished for his zeal in advocating the importance of lectures on military surgery, as a means by which medical officers, who have been serving on foreign stations, may "recover their lost ground, and qualify themselves to take their place among the well-informed members of the profession." It seems, however, to have escaped his notice, that these officers, even after many years' absence from their native land, rarely obtain, on their return, more than one or two months leave of absence, provided they are fit for duty, and are usually more disposed to spend that with their friends in the country, than to resort to the "Metropolitan cities of the Empire," although, by so doing, they might obtain "the most recent, the most satisfactory, and the most authentic information on the progress of science, and on the improvements which have taken place during their absence." We feel bound in justice to add, that we understand his exertions to stimulate the emulative industry of his class—a most important means of exciting to professional improvement—have been such as to deserve the highest praise, and are well worthy of imitation.

The Medical Board at Calcutta, in 1818, issued a circular, "in conformity with the orders of Government," in which they state;—"It has been a frequent subject of regret to the medical world, that wide and rich as is the field in India for the cultivation of physical science and of pathology, the labour hitherto employed in it, even in comparison with what has been bestowed on far less promising spots, has been negligent and irregular; and the product presented to the public scanty, and in the extreme unprofitable. Whilst in almost every other portion of the civilised world scientific objects have been pursued with energy and success, and every successive year has added largely to the previous stock of medical knowledge, India has scarcely given birth to a single important discovery, or publication of acknowledged merit on professional subjects, since the first establishment of the British interests in this quarter."

We do not intend to examine how far these observations may still apply to the Medical Department of the Company's Service, or whether the officers of the Queen's Army, and of the Navy, may not, in some degree, merit a similar censure. We believe they are not deficient in point of professional zeal and intelligence, but hitherto the fruits of their experience have in a great measure been confined within a narrow sphere, and thus, whilst the medical world has been deprived of the principal end and use of past observation and experience, that of serving as a guide for the future, the junior branches of the service have been shut out from benefiting by the fund of knowledge acquired by their seniors. The following remarks, bearing upon this point, are quoted from the important and authoritative circular above noticed:—"The individuals composing this respectable class (the Medical Department) have ever been remarkable for the diligent and able performance of their duties. They would seem to be, by previous regular education, and by subsequent large acquaintance with the diseases usually prevalent in hot climates, peculiarly qualified to impart useful information to their less experienced brethren." "The true nature and treatment of fever, bowel complaints, and the other classes of disorders common to India with other regions, are yet very imperfectly understood, and their investigation, while it lies peculiarly open to practitioners in this country, would be hailed by their brethren in Europe, as opening a path that must lead to conclusions important and interesting to all. The various species of cutaneous diseases, elephantiasis, and other maladies almost peculiar to warm countries, present a wide and nearly untrodden field to the curiosity of future investigators."

The history of the arts and sciences in this country, affords an instructive proof of the advantages to be derived from exciting a feeling of emulation in any body of men. One of the most striking examples of this may be found in the results that have followed the exertions of the Highland Society of Scotland, which, by offering liberal premiums for essays and memoirs on subjects connected with agriculture, successfully stimulated individual industry, and in a few years produced a complete revolution in that science. With a view to the application of the same principle to medicine, the following advertisement was issued at Madras :

" Fort St. George Gazette, No. 38, dated 12th May, 1832, Saturday.

ADVERTISEMENT.

*Medical Prize Essay.*

" With a view to the advancement of medical science and to communicate useful knowledge, the Medical Board, under the sanction of Government, announce to the Medical Officers under this Presidency, whether of His Majesty's or the Honorable Company's Service, that a prize of Rupees 500, or a gold medal of that value, with a suitable inscription, will be awarded in the course of the year 1833, to the best dissertation on each of the following subjects,

" 1. On the disease called 'Beriberi,'

" 2. On Rheumatism and the Neuralgic Affection, occasionally a sequela of it, which is termed among the natives 'Burning in the Feet.' "

Four essays on " Beriberi," and three on " Rheumatism and Burning in the Feet," were received, all of them possessing very considerable merit, the prize being awarded to Dr. J. G. Malcolmson, of the Madras European Regiment, whose essay on Beriberi contained a very able and laborious investigation into the causes, nature, and treatment of that disease, eminently calculated to impart just views of the former, and to render the latter more discriminating and successful. His essay on Rheumatism was equally satisfactory. Both papers were published at the expense of Government, and distributed among the medical officers of the Madras establishment.

The late Sir Gilbert Blane left a sum of money, the interest of which was to be appropriated to the purchase of a gold medal, for the Medical Officer in the Navy, who, during each year, shall have kept his medical journals in the most satisfactory manner. The establishment of this prize has been followed by a marked improvement in this particular department of the officer's duty, and throughout the service there is a great ambition to obtain this honorable distinction.

Considering the success which has so eminently attended all these experiments, we are much disposed to think, that the liberality of Government would be more beneficially exercised in awarding premiums for essays and memoirs, than in endowing professorships of military surgery. Abundant subjects for these might be found in the diseases peculiar to our various colonies and dependencies, and in those incident to a naval or military life. For instance, some of the following might be selected :— A Medical History of the Chinese War, or of the War in Affghanistan ; On the Endemic Diseases of the East; On the Epidemics of the East; On the Fevers of the West Indies ; On the Geographical Distribution of Diseases ; On the Means of preserving the Health of Soldiers and Sailors,

&c. Were Government to follow the example of the Madras Board, and publish the prize essays for gratuitous distribution to the officers of both services, the advantages of this scheme would be much enhanced. The inducements to exertion would be increased by the certainty of the successful work being extensively circulated among those who would be most likely to appreciate it, while, at the same time, the officers of the medical department would be periodically supplied with the most recent and complete information on points of importance connected with their profession. But another and even more desirable end would probably be attained; officers would be induced, from the commencement of their career, to collect information whenever an opportunity should present itself, in the hope that, at some future period, it might be made available for this honorable competition, and would thus spend in the acquisition of useful knowledge, many of those hours, which, we fear, are at present spent in the billiard-room, or dissipated in some of those ingenious methods devised, both in the Army and Navy, to kill time. In preparing these essays, it is not the mere collection of information on the subjects of them that is gained, but, in his researches, the writer adds to his knowledge on all the collateral branches of science, and however much the profession may profit by his labours, there can be no doubt that the greatest share of benefit accrues to himself. Viewing it then as a most important means of individual improvement, and consequently of increasing the efficiency of the Department, we most earnestly desire to see some scheme of this nature in operation. Sir Robert Peel seems well aware of the advantages derived from competition among the Farmers, and has recently, in his own county, promised to aid them in their experiments in agriculture. Let him try an experiment in another branch of science, and establish prizes to be competed for annually by the Medical Officers of the Army and Navy, and we may safely predict, he will thereby confer on the United Service a great and lasting benefit at a trifling expense to the country.

